# Bacterial community composition, quantification of antibiotic resistance genes and antibiotic residues in wastewater treatment plant and receiving rivers

**DOI:** 10.3389/fmicb.2025.1635253

**Published:** 2025-10-27

**Authors:** Karabo Tsholo, Lesego Molale-Tom, Suranie Horn, Cornelius Carlos Bezuidenhout

**Affiliations:** ^1^Unit for Environmental Science and Management Microbiology, North-West University, Potchefstroom, South Africa; ^2^Faculty of Health Science, Occupational Hygiene and Health Research Initiative (OHHRI), North-West University, Potchefstroom, South Africa

**Keywords:** antibiotics, bacterial communities, fluconazole, high-throughput 16S rRNA sequencing, physicochemical parameters, real-time PCR, wastewater treatment plants

## Abstract

Antibiotic resistance was, until recently, primarily documented as a clinical phenomenon, with limited consideration given to environmental settings in policymaking. Although literature has demonstrated the presence of antibiotic-resistant bacteria in water environments, there is limited information on the concentrations of antibiotic resistance genes (ARGs) and antimicrobial residues, particularly in sub-Saharan Africa. Hence, this study aimed to determine the concentrations of ARGs and antimicrobial residues in urban wastewater effluents and receiving waters in North West Province, South Africa. The physicochemical parameters of the water samples were determined, and the ARGs were screened and quantified using end-point and real-time PCR. Chemical analysis was performed to quantify the concentrations of antibiotics and fluconazole. High-throughput 16S rRNA sequencing was conducted to identify and profile bacterial communities. Correlations between bacterial communities and environmental parameters were determined. Physicochemical parameters indicate that the water quality from wastewater effluent and receiving waters poses no concern for livestock use. However, most were correlated with the presence of distinct microbial communities, of which Proteobacteria and Bacteroidota were the dominant groups. Elevated ARG levels, inducing multidrug resistance, were reported in river water, indicating the longevity and transfer of ARGs in the environment. Notably, river water and wastewater effluent were more contaminated with ampicillin compared to other antimicrobials. The presence of antimicrobials may select for the development of antimicrobial resistance. Despite the widespread presence of ARGs and antimicrobial residues in water environments, these contaminants are not routinely monitored or regulated. The presence of these contaminants in water poses human and ecological risks.

## 1 Introduction

Wastewater treatment plants (WWTPs) are systems designed to prevent contaminants from entering receiving water bodies (Luo et al., 2025). However, they are not designed to eliminate all pollutants and pathogens, for example, antibiotic-resistant bacteria, antibiotic-resistant genes (ARGs) and antimicrobial residues (Chamkal et al., 2021; Rodríguez et al., 2021; Späth et al., 2021; Luo et al., 2025). Due to incomplete metabolism in humans, many antimicrobials are excreted as metabolites and unchanged parent compounds via feces and urine, leading to their spread in WWTPs and are subsequently released into the environment (Chamkal et al., 2021). Apart from the incapacity of WWTPs to eliminate these compounds, the Department of Water and Sanitation (DWS, 2022) noted that most South African WWTPs are currently in a critical condition. This hinders the reusability of reclaimed water since discharged WWTP effluent may pollute receiving freshwater bodies with these contaminants (Massé et al., 2014; Freeman et al., 2018). Subinhibitory concentrations of antimicrobials in the aquatic environment may act as selective pressures that promote the emergence of antibiotic resistance (Boxall et al., 2004; Massé et al., 2014). Furthermore, physicochemical parameters and seasonal factors may be favorable for the proliferation of ARGs (Rodríguez et al., 2021). The ARGs are shared via horizontal gene transfer (HGT) by opportunistic and pathogenic bacteria (Ateba et al., 2020; Maguvu et al., 2020). Despite this, ARGs and antimicrobials are not monitored or regulated in water environments in South Africa, where antimicrobials are widely used for both human and animal purposes (NDoH, 2018).

Moreover, in South Africa, a gap exists in the literature regarding the abundance of ARGs in water environments (Coertze and Bezuidenhout, 2020). Furthermore, antimicrobials in the environment are underexplored, overlooking the potential risk to human and ecological health (Assress et al., 2020; Kairigo et al., 2020; Chamkal et al., 2021). These limitations ultimately contribute to the already heightened antibiotic resistance, complicating antibiotic therapy (Ateba et al., 2020). Therefore, this study aimed to assess antimicrobial residue concentrations and ARG patterns in WWTP effluent and rivers across two regions of the North West Province, South Africa. Additionally, high-throughput 16S rRNA sequencing was employed to identify bacterial communities associated with physicochemical properties, antibiotic resistance genes, and antimicrobial residues. The present study specifically intends to address the following research question: What is the prevalence and distribution of ARGs, antimicrobial residues and bacterial communities in WWTP effluent and surrounding rivers?

## 2 Methods

### 2.1 Study areas and sample collection

The municipality permitted the study of these samples on the condition of anonymity. The study areas, North West-E (NW-E) and North West-C (NW-C), are situated within the same municipality in the North West Province, South Africa. The NW-E and NW-C regions have a semi-arid climate, characterized by dry winters and wet summers. The rivers where NW-E and NW-C are situated form part of an interconnected fluvial system of the province. These rivers are used for domestic, agricultural, recreational, industrial and ecosystem services. The sectors in which these rivers serve may lead to point and non-point source pollution, which can affect water quality. Additionally, upstream mining activities in the NW-E catchment may also impact water quality. The study areas’ WWTP capacities, processes, dam capacity, and population sizes are provided in Table 1.

**Table 1:** Description of the NW-E and NW-C study areas.

Sites	Plant capacity (Mℓ/day)	Treatment processes	Dam capacity (M^3^)	Population size	Households serviced
NW-E	45	Biofilters—activated sludge—chlorination	2.03 × 10^6^	162,762	52,537
NW-C	3	Screening—drying bed—biofilters—clarification—chlorination	5.67 × 10^6^	56,702	14,562

Water samples were collected in upstream rivers, WWTP effluents and downstream points (NW-E = river and NW-C = maturation pond). Sample collection started from October 2020 to March 2022. Water samples were collected in 2020 (October, November and December), 2021 (February, March, April, July, August, September, October and December) and 2022 (February and March). These sample dates represent the wet season (October to March) and the dry season (April to September) of the North West Province. Grab samples were collected in sterile (autoclaved) 1 L bottles (Duran Schott, Germany) to analyze bacterial community composition and ARGs. Acid-washed 1 L bottles (free from chemical contaminants) were used to collect water to analyze antimicrobial residues. Samples were collected in up to three replicates from each sampling point. Water samples were stored and transported on ice to the laboratory for analysis, which commenced within 24 h of sampling. The municipality permitted the study of these samples on the condition of anonymity.

### 2.2 Physicochemical parameters

Temperature, total dissolved solids (TDSs), pH, and salinity were measured *in situ* with a portable multimeter (PCSTestr 35, Eutech Instruments Pte Ltd., Singapore). In the laboratory, phosphorus (HACH method 8,178), nitrite (HACH method 8,153), nitrate (HACH method 8,039) and chemical oxygen demand (COD; HACH method 8,000) levels were measured by using a Hach Lange DR 2800 spectrophotometer (Hach Company, United States), according to its manual protocol. Where applicable, measured physicochemical parameters in NW-E and NW-C were compared to the Department of Water Affairs and Forestry’s (DWAF) South African water quality guidelines (SAWQG) (DWAF, 1996a,b) for agricultural use: irrigation and livestock watering. Physicochemical parameters were measured from water samples from December 2020 until March 2022.

### 2.3 Filtration and deoxyribonucleic acid isolation

One litre of the sample was filtered using a sterile 0.45 μm mixed cellulose ester filter membrane (PALL Life Sciences, Mexico). The environmental deoxyribonucleic acid (eDNA), captured cellular DNA on filters, was isolated using a PowerWater kit (Qiagen, Netherlands), according to the manufacturer’s protocol. The eDNA was quantified and the quality was determined using a Nanodrop 1,000 (Thermo Fischer Scientific, United States). This was performed on samples collected from October 2020 until March 2022.

#### 2.3.1 Library preparation and amplicon sequencing

Samples (DNA) collected in December 2021 and March 2022 were subjected to next-generation sequencing. The 16S rRNA barcode sequencing library preparation was performed according to the manufacturer’s protocol (Illumina, USA). The 16S rRNA gene-specific sequences (16S Amplicon PCR Forward Primer = 5’ TCG TCG GCA GCG TCA GAT GTG TAT AAG AGA CAG CCT ACG GGN GGC WGC AG and 16S Amplicon PCR Reverse Primer = 5’ GTC TCG TGG GCT CGG AGA TGT GTA TAA GAG ACA GGA CTA CHV GGG TAT CTA ATC C) were used to target the V3 and V4 region. Briefly, locus-specific primers were appended with Illumina forward (5’TCG TCG GCA GCG TCA GAT GTG TAT AAG AGA CAG) and reverse overhang adapter (5’GTC TCG TGG GCT CGG AGA TGT GTA TAA GAG ACA G) nucleotide sequences for sample identification as per the manufacturer’s protocol.

There were two PCR stages in which their amplicons were purified using Agencourt AMPure XP beads (Beckman Coulter Genomics, USA). Amplicon PCR conditions were as follows: initial denaturation at 95 °C for 3 min, 25 cycles of denaturation at 95 °C for 30 s, annealing at 55 °C for 55 s, elongation at 72 °C for 30 s and final extension at 72 °C for 5 °C minutes. Index PCR conditions were as follows: initial denaturation at 95 °C for 3 min, 8 cycles of denaturation at 95 °C for 30 s, annealing at 55 °C for 30 s, elongation at 72 °C for 30 s and final extension at 72 °C for 5 °C min. The Qubit 3.0 fluorometer (ThermoFisher, USA) was used to quantify amplicons after the second PCR. The pair-end (2 × 300 bp) sequencing was performed in-house on the Illumina MiSeq platform (Illumina, USA).

#### 2.3.2 16S rRNA amplicon data processing and taxonomic assignment

Raw data sequences were analyzed using the QIIME 2 pipeline (version 2024.10.1) for demultiplexing, removing poor-quality sequences, and trimming primer sequences and adaptors using the DADA2 plugin. However, only the forward reads were subjected to the follow-up analyses due to the poor quality of the reverse reads. The SILVA 138.99 ribosomal RNA gene database^1^ was used to cluster and classify operational taxonomic units (OTUs) with a 97% identity threshold (Bolyen et al., 2019).

The OTUs data were exported to R Studio (2024.12.1) for further analyses. Stacked column plots illustrating phyla- and genera-level changes in bacterial community composition of WWTP effluent, upstream rivers, and downstream points at NW-C and NW-E were created. A box plot showing the alpha diversity (ACE, B-Chao1, Shannon, and Simpson) for each sample was done, and a Kruskal-Wallis test was performed to establish whether there were significant differences (*p*-value < 0.05).

#### 2.3.3 Screening of antibiotic resistance genes

All samples were subjected to end-point polymerase chain reaction (PCR) (Thermo Fisher Scientific, UK), which was conducted using the Techne^™^ PCRmax Alpha Cycler 1 to screen ARGs listed in Supplementary Tables S1, S2. These tables also represent the primer sequences, size and cycling conditions. Most featured ARGs, such as six plasmid-mediated (p) AmpC (DHA, FOX, ACC, CIT, MOX, and EBC), *bla_TEM_* and *ampC,* are clinically relevant genes associated with *β*-lactam resistance (Coertze and Bezuidenhout, 2020). The *ermB* and *ermF* genes, associated with macrolide resistance, are commonly found in environmental and clinical settings (Chen et al., 2015; Quaik et al., 2020). The *sul1* and *sul2* genes, associated with sulfonamide resistance, are widespread in environmental settings due to their association with mobile genetic elements (Freeman et al., 2018). The class I integron integrase gene *intI1* was chosen as a marker for HGT potential (Thakali et al., 2020). Each reaction mixture consisted of 25 μL in volume, containing 2 μL of bacterial DNA template, 12.5 μL DreamTaq PCR master mix (Thermo Scientific, US), 1 μL of each oligonucleotide (forward and reverse) primer and 8.5 μL nuclease-free water (Fermentas Life Sciences, US). Negative and positive controls were included in each reaction.

#### 2.3.4 Quantification of 16S rRNA and antibiotic resistance genes

Absolute real-time PCR using QuantStudio^™^ 3 platform (Applied Biosystems, Thermo Fisher Scientific, USA) was done to quantify 16S rRNA, pAmpCs, *sul1* and *intI1* genes. The quantification of 16S rRNA and ARGs each involved a 10 μL reaction mixture, following the manufacturer’s protocol for the Power Up SYBR Green Master Mix (Applied Biosystems, Thermo Fisher Scientific, Lithuania). Negative and positive controls were included in each reaction. Supplementary Table S3 presents the primer sequences and cycling conditions of 16S rRNA, *sul1* and *intI1* genes. The following FAM fluorescent dyes TaqMan gene expression assays (Thermo Fisher Scientific, USA) were used for quantification of pAmpCs: Ba04646120_s1 (DHA gene), Ba04646126_s1 (FOX gene), Ba04646144_s1 (ACC gene), Ba04646135_s1 (CIT gene), Ba04646156_s1 (MOX gene), and Ba04646124_s1 (EBC gene). According to the standard TaqMan protocol, the exact probe sequences are proprietary and not publicly disclosed. The ARG abundance was divided by the concentration of 16S rRNA to represent the abundance as gene copies per 16S rRNA.

### 2.4 Extraction of antimicrobials

Antibiotics (ampicillin, ciprofloxacin, trimethoprim and sulfamethoxazole) and antifungal (fluconazole) from WWTP effluent and surrounding water were quantified. The selected antibiotics belong to the access (trimethoprim) and watch categories (ciprofloxacin, ampicillin, sulfamethoxazole) (WHO, 2021). Antimicrobial resistance extends beyond antibiotics; thus, antifungal fluconazole was selected (Monapathi et al., 2021). In South Africa, fluconazole is commonly prescribed to patients living with HIV to treat potential pathogenic fungi (NDoH, 2020).

Before the solid-phase extraction (SPE), preparations of one-litre samples involved adding 1 g/L ethylenediaminetetraacetic acid disodium salt dihydrate (Na_2_EDTA) (Merck, United States) and adjusting its pH to 2 by using 32% HCl (Rochelle Chemicals, RSA). The target compounds (ampicillin, ciprofloxacin, trimethoprim, sulfamethoxazole and fluconazole) were extracted from the samples using HLB-H disks and an automated SPE-DEX system (Horizon Technology, Salem, NH, USA). The disks were pre-conditioned with methanol, followed by ultra-high-purity water and ultra-high-purity water with a pH of 2. The water samples were then automatically subjected to extraction with the SPE-DEX. The disks were air-dried for 15 min and the analytes were eluted with methanol and acetone/methanol (1:1). A gentle stream of nitrogen gas was used to evaporate the eluate within a water bath of 37 °C and then reconstituted with 1 mL of methanol- Milli-Q water (1:1) and 0.1% formic acid (Honeywell, United States).

#### 2.4.1 UHPLC-QTOF/MS analysis of antimicrobial residues

An ultra-high-performance liquid chromatography system coupled to a quadrupole time-of-flight mass spectrometry (UHPLC-QTOF/MS) was used to quantify the antimicrobials. A Poroshell Bonus-RP 120 (2.1 × 100 mm; 2.7 μm) column (Agilent Technologies, USA) was used to separate the target compounds. The ionization was performed using positive electrospray ionization enhanced with Agilent Jet Stream technology. The separation of compounds was conducted in gradient mode using a mobile phase consisting of 0.1% (v/v) formic acid in Milli-Q water (A) and 0.1% (v/v) formic acid in acetonitrile (B). Elution program was as follows; 5% B in the first 2 min at 0.2 mL min^−1^, 15% B from 2 to 5.55 min at 0.4 mL min^−1^, 20% B from 5.55 to 11.10 min at 0.2 mL min^−1^, 25% from 11.10 to 13.15 min at 0.6 mL min^−1^, 35% from 13.15 to 17.20 min at 0.2 mL min^−1^, 100% B from 17.20 to 20.0 min at 0.2 mL min^−1^ and 5% from 20.0 to 22.0 min at 0.2 mL min^−1^. The QTOF settings were as follows: 250 °C drying gas, 8 L/min drying gas flow, 35 psig nebulizer pressure, 250 °C sheath gas temperature, and 5.5 L/min sheath gas flow.

#### 2.4.2 Method validation

The matrix effects were considered by preparing an external matrix-matched calibration curve, in which the matrix was spiked in triplicate with known concentrations of target compounds (0 to 10 μg/L) using eight standard curve points. The linearity, represented as R2, was also determined. The limit of detection (LOD) and limit of quantification (LOQ) were calculated by using the following formula:


LOD=3∗ab

LOQ=10∗ab

Where: a = standard deviation; b = slope.

The method’s accuracy was determined by spiking the matrix in triplicate with known low, medium and high concentrations to calculate the recovery rates of the target compounds after extraction. The intra-day precision was calculated as the relative standard deviation of six replicates analyzed daily for 3 days. The inter-day precision was calculated as the relative standard deviation of six replicates analyzed across 3 days (Späth et al., 2021).

#### 2.4.3 Risk quotient

The concentrations of fluconazole and antibiotic residues were used to calculate the risk quotient (RQ), as described by Coors et al. (2018) and Tell et al. (2019). The RQ ≥ 1 indicates high risk, < 1 medium risk and < 0.1 low risk to the environment. However, risk profiling for ARGs was not conducted due to the lack of a standardized method. The following equation shows the calculation of antimicrobial RQ:


$$\mathrm{RQ}=\frac{\mathrm{Con}}{\text{PNEC}}$$

where: RQ, risk quotient; con = antibiotic or fluconazole concentrations; PNEC-ENV, environmental predicted no-effect concentration.

### 2.5 Statistical and data analyses

R Studio was used to establish whether there were significant differences in the measured physicochemical parameters, fluconazole, and antibiotic residues between sampling sites. Antimicrobial residue concentrations < LOQ were set to zero to standardize data handling for statistical analysis. The Shapiro–Wilk test was performed to determine the normality of the data. The failure of the normality test necessitated data transformation using the square root or log transformation. The data that was not transformed was subjected to a Kruskal-Wallis test to determine significant differences (*p*-value < 0.05) between sampling sites with more than two independent variables. Student’s *t*-test or ANOVA was performed for normally distributed data. The Tukey post-hoc test was performed with the *p*-value adjusted using the Bonferroni method to establish differences among sampling sites for the statistically significant group.

Moreover, R Studio was used to create a correlation plot using the Pearson method to determine whether there were significant differences (*p*-value < 0.05) between the correlated groups. The correlation was between bacterial communities, physicochemical parameters, ARGs, fluconazole, and antibiotic residues. Significant levels (*p*-value < 0.05) are represented by an asterisk (*). The ARGs were correlated using the end-point PCR results, as suggested by Coertze and Bezuidenhout (2020). Furthermore, R Studio was used to create a line plot for physicochemical parameters. Heatmaps showing the detected ARGs and RQs of antimicrobial residues were constructed using GraphPad Prism (version 9.0.1, 2019).

### 2.6 Analytical method validation

The method was validated by plotting standard curves to determine the slope, coefficient of determination (*R^2^*), and efficiency of each gene. Serial dilutions (1:10) with known abundance isolated from the American Type Culture Collection were prepared. The serial dilutions were performed in triplicate for the five standard curve points. The efficiency (E) and slope (s) were calculated using the following equation (Coertze and Bezuidenhout, 2020).


$$E=10^{-1/s}-1$$

Supplementary Table S4 shows the method validation parameters for 16S rRNA and ARGs. The y-intercept and Ct cut off of genes performed using the TaqMan assay were higher than those of SYBR Green assays. The data showed that the coefficient of determination (*R*^2^) was above 0.98, with an efficiency range of 95 to 105% and a slope range of −3.46 and −3.22. These data are reliable for determining the unknown abundance of 16S rRNA and ARGs using the linear equation in water environments (Coertze and Bezuidenhout, 2020; Späth et al., 2021).

Furthermore, Supplementary Table S5 shows that fluconazole and antibiotics were successfully analyzed, with *R*^2^ above 0.99. Overall, the RSD of the matrix was below 20%, and the recoveries ranged from 90 to 102%. Mass spectrometry and retention time of each target compound are also shown in Supplementary Table S5.

## 3 Results

### 3.1 Physicochemical parameters analysis

**Figure 1:**
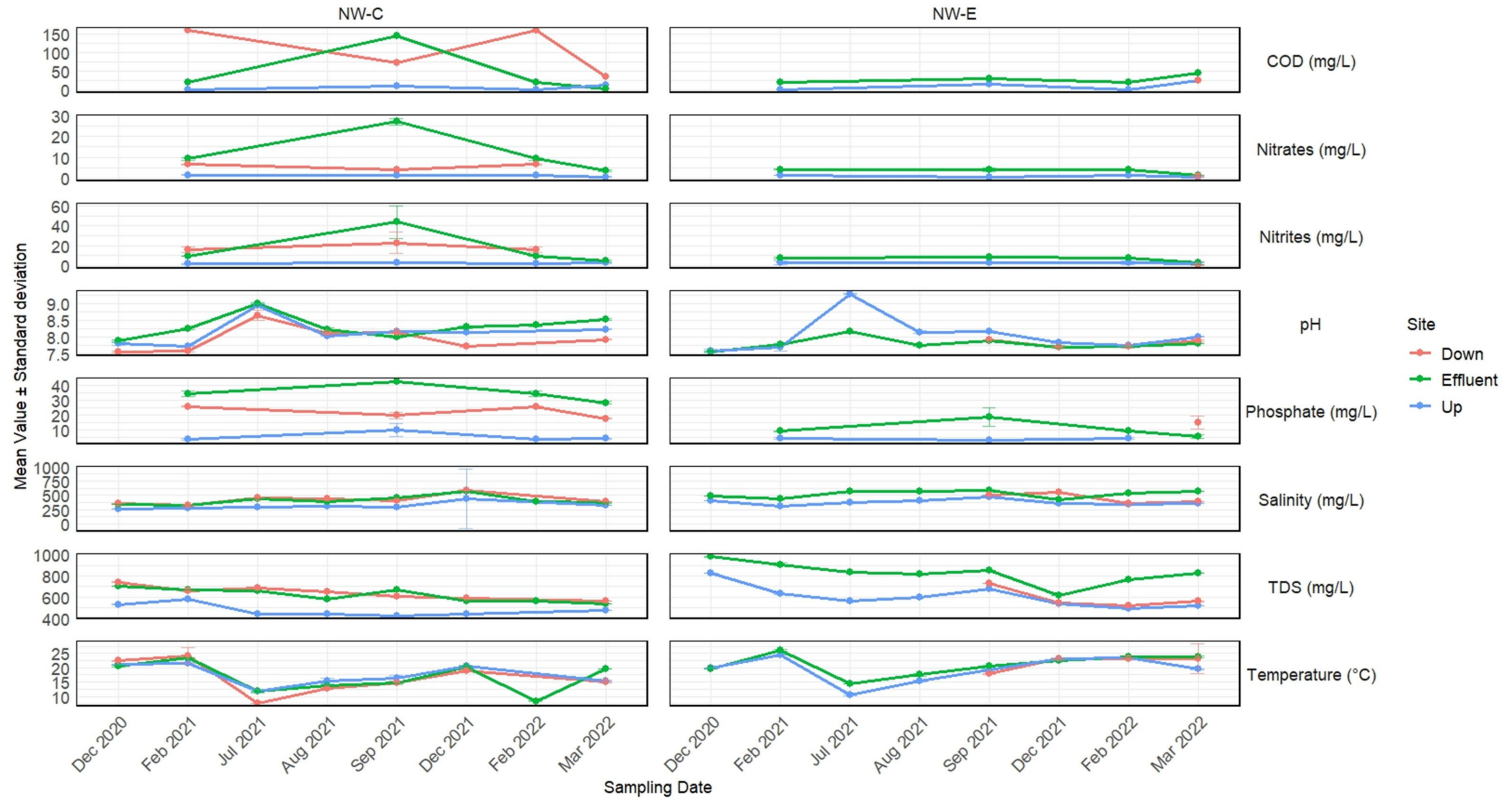
Physicochemical parameters with standard deviations measured in 2020, 2021 and 2022 in the upstream rivers, wastewater effluent and downstream points at NW-E and NW-C.

Physicochemical parameters of NW-E and NW-C water quality are depicted Figure 1. The interpretation of results in this section is based on mean values of each physicochemical parameter per location from Figure 1. The general trend for physicochemical parameters was that TDS, salinity, nitrate and COD levels were higher in the downstream river at NW-E after blending with the wastewater effluent. The levels were as follows: Upstream river (TDS = 606.25 mg/L, salinity = 378.00 mg/L, nitrate = 0.88 mg/L and COD = 13.33 mg/L), wastewater effluent (TDS = 824.88 mg/L, salinity = 525.13 mg/L, nitrate = 3.28 mg/L and 32.00 mg/L) and downstream river (TDS = 613.33 mg/L, salinity = 480.00 mg/L, nitrate = 1.07 mg/L and COD = 25.00 mg/L). Furthermore, TDS, salinity and nitrite levels differed significantly (*p*-value < 0.05) between WWTP effluent, downstream and upstream rivers.

In NW-E, phosphate levels were higher in the downstream river (14.95 mg/L) compared to the WWTP effluent (11.06 mg/L) and upstream river (3.53 mg/L), with levels in WWTP effluent significantly different (*p*-value < 0.05) from those in the downstream and upstream rivers. Furthermore, the downstream river recorded lower nitrite levels (0.00 mg/L), with significant differences (*p*-value < 0.05) between the WWTP effluent and the upstream river. As anticipated, high and low temperatures were recorded in the winter and summer months, respectively.

Generally, lower physicochemical levels, except pH and temperature, were measured in the upstream river in NW-C compared to the WWTP effluent and downstream maturation pond (Figure 1). Moreover, the physicochemical levels of the upstream river, except for pH and temperature, differed significantly (*p*-value < 0.05) from those measured in the WWTP effluent and the downstream maturation pond. Higher pH, phosphate and nitrate levels were measured in the WWTP effluent. However, the TDS, salinity, nitrite and COD levels were higher in WWTP effluent compared to the downstream maturation pond. Significant differences (*p*-value < 0.05) were recorded between the downstream maturation pond and WWTP effluent for pH and phosphate levels.

Overall, the physicochemical parameters (TDS, salinity and nitrate) for water samples at NW-E and NW-C were within the recommended limits of DWAF (1996a,b) for agricultural use: Irrigation and livestock water. However, pH > 8.4 in Table 4 was on the higher end of this spectrum.

### 3.2 Bacterial community composition

The 16S rRNA gene metabarcoding was performed on samples collected in December 2021 and March 2022. Only the forward reads were subjected to downstream analysis. After trimming low-quality reads (including adapters and primers) and denoising data (for error correction and chimera removal), the number of reads ranged from 65,391 to 91,138 per sample. Furthermore, reads were rarefied to normalize sequence depth for diversity analysis. The raw sequencing data generated in the present study were submitted to the National Center for Biotechnology Information (NCBI) Sequence Read Archive (SRA) under Bio Project accession number PRJNA1266490. Sample IDs, along with their corresponding accession numbers, are listed in Supplementary Table S6.

**Figure 2:**
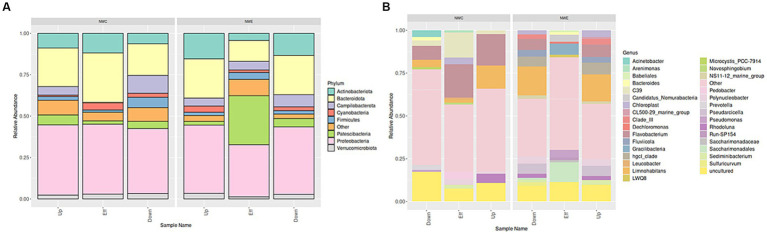
Stacked column plots illustrating **(A)** phyla- and **(B)** genera-level changes in bacterial community composition of WWTP effluent, upstream rivers, and downstream points at NW-C and NW-E, with a relative abundance >2%. Up, upstream river; Effluent, WWTP effluent; down, downstream maturation pond.

Figure 2 illustrates the phylum- and genus-level composition of the bacterial community, with a relative abundance of ≥ 2%. The category “other” represents bacterial communities with a relative abundance of < 2%. In NW-E, the five phyla with high relative abundance were Proteobacteria, Bacteroidota, Actinobacteriota, Patescibacteria, and Campilobacterota, as shown in Figure 2A. In NW-C, the trend was as follows: Proteobacteria > Bacteroidota > Other > Actinobacteriota > Patescibacteria. A total of 33 genera, including “other,” were present in NW-E and NW-C as shown in Figure 2B. In NW-C, the five genera with high relative *abundance are Flavobacterium, uncultured, C39*, and *Polynucleobacter*. In NW-C, the trend was as follows: C39, uncultured, *Flavobacterium, Saccharimonadales* and hgcI_clade.

**Figure 3:**
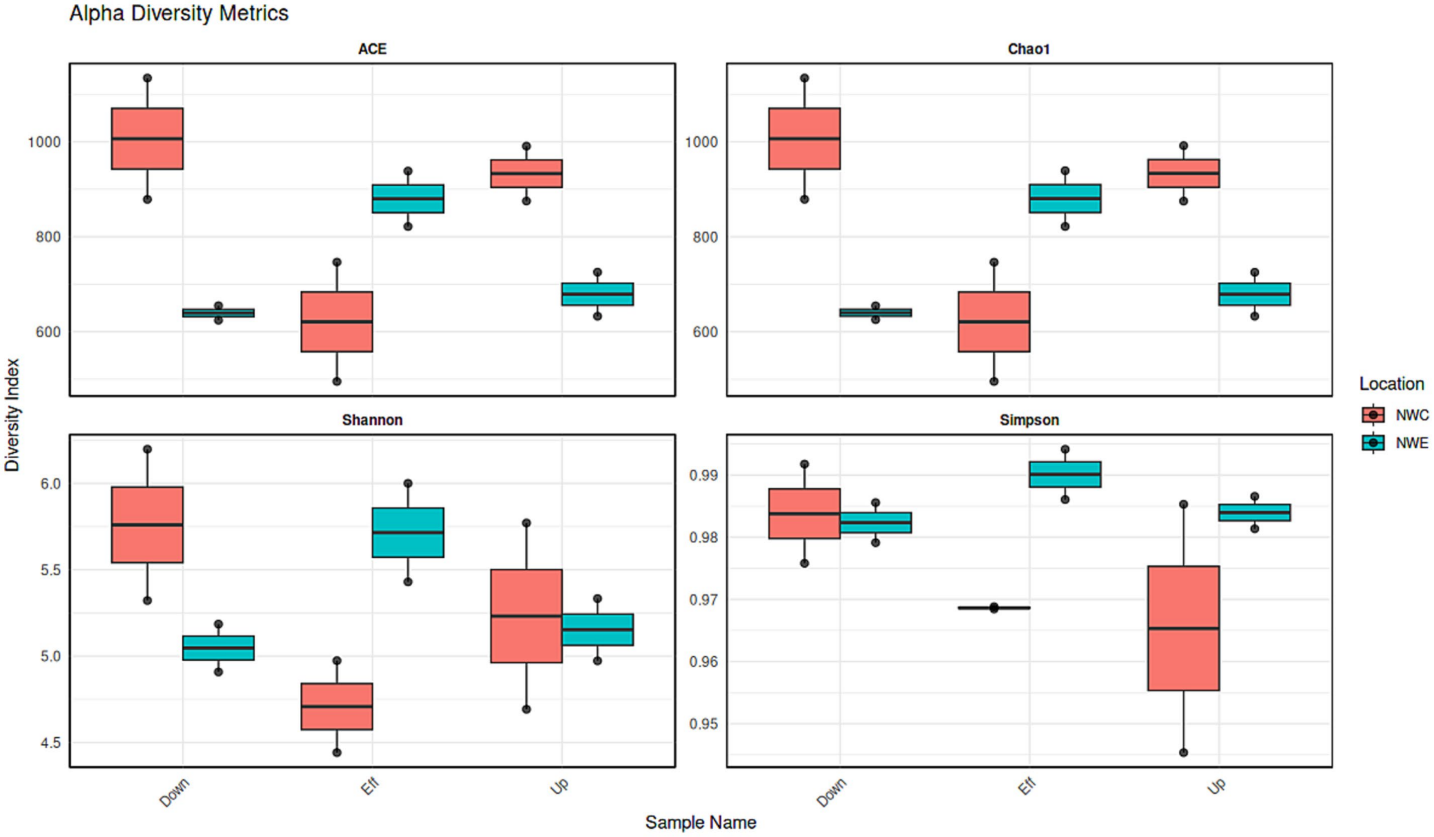
Bacterial alpha diversity indices (ACE, B-Chao1, Shannon and Simpson) for upstream rivers, wastewater effluent and downstream points at NW-E and NW-C. Up, upstream river; Effluent, WWTP effluent; down, downstream maturation pond.

Figure 3 depicts the alpha diversity with a focus on the Simpson index (species dominance), Shannon index (species diversity and evenness), Chao1 and ACE estimator (richness). In NW-E, WWTP effluent had higher species dominance, richness, evenness and diversity. In NW-C, the downstream maturation pond displayed more species dominance, diversity, richness and evenness than the upstream river and WWTP effluent. However, a Kruskal-Wallis test shows no significant differences (*p*-value > 0.05) for upstream rivers, wastewater effluent, and downstream points for each alpha diversity test.

### 3.3 Screening of antibiotic resistance genes

**Figure 4:**
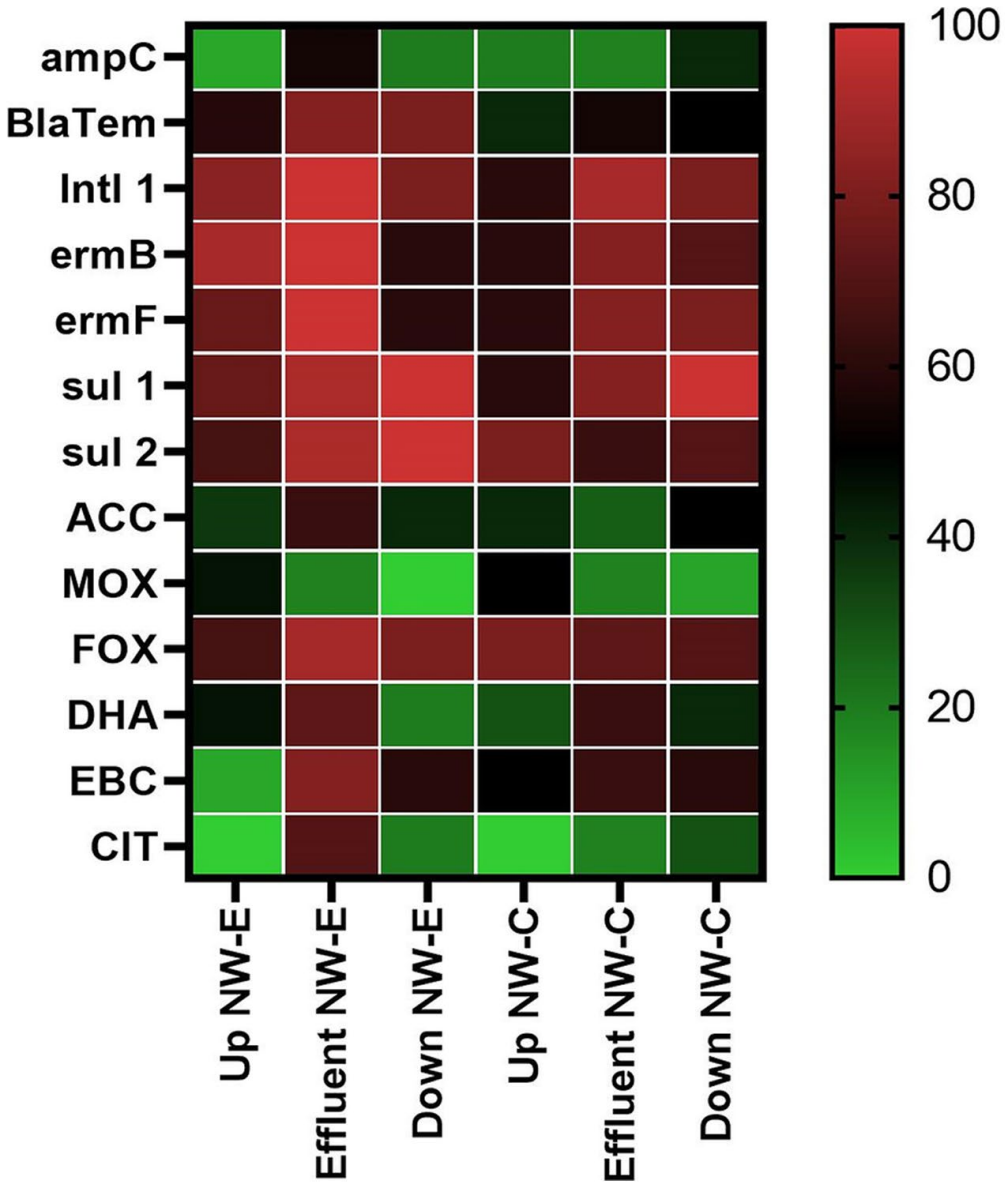
Heatmap of the detected ARGs in the upstream rivers, WWTP effluent and downstream points at NW-E and NW-C. Up, upstream river; Effluent, WWTP effluent; down, downstream maturation pond.

In the NW-E system, *ampC*, *bla_TEM_*, *int1*, *ermB*, *ermF*, ACC, FOX, DHA, EBC, and CIT genes were detected in higher percentages of analyzed samples in the WWTP effluent compared to the upstream and downstream river samples (Figure 4). However, the *sul1* and *sul2* genes were detected in all downstream river samples. Abnormalities were recorded for the MOX gene, with a higher proportion of samples from the upstream river sites (45.45%). The CIT and MOX genes were undetected in upstream and downstream river samples.

In NW-C, the detection of ARGs fluctuated between the upstream river, WWTP effluent and downstream maturation pond (Figure 4). The upstream river had more samples positive for *sul2* (80%), MOX (50%), and FOX (80%) genes compared to the WWTP effluent and downstream maturation pond. The downstream maturation pond recorded a higher percentage of samples positive for *ampC* (40%), *sul1* (100%), CIT (30%), and ACC (50%) genes. More of the WWTP effluent samples were positive for *bla_TEM_* (54.55%)*, intI1* (90.91%), *ermB* (81.82%), *ermF* (81.82%), DHA (63.64%), and EBC (63.64%) genes. The CIT gene was not detected in the upstream river samples.

### 3.4 Quantification of antibiotic resistance genes

The ARG abundance and standard deviations are shown in Table 2. In NW-C, ACC abundance was higher in the upstream river (1.97 × 10^−1^ gene copies/16S rRNA) than in WWTP effluent (8.34 × 10^−3^ gene copies/16S rRNA) and downstream maturation pond (8.61 × 10^−2^ gene copies/16S rRNA). Furthermore, the abundance in the maturation pond was higher than in the WWTP effluent. In NW-E, the ACC concentration was higher in the WWTP effluent (2.90 × 10^−1^ gene copies/16S rRNA) as compared to the upstream (1.00 × 10^−1^ gene copies/16S rRNA) and downstream rivers (6.11 × 10^−2^ gene copies/16S rRNA). FOX and *intI1* abundance was higher in the upstream river than in the WWTP effluent and downstream river in NW-E (Table 2). In NW-C, a similar trend was observed for the FOX gene, where the abundance of gene copies was higher in the upstream river (4.06 × 10^0^ gene copies/16S rRNA) compared to the WWTP effluent (7.57 × 10^−1^ gene copies/16S rRNA) and downstream maturation pond (1.38 × 10^0^ gene copies/16S rRNA). However, this was not the case for the *intI1* gene in NW-C. Downstream maturation pond (5.38 × 10^−4^ gene copies/16S rRNA) had higher *intI1* abundance than WWTP effluent (4.09 × 10^−4^ gene copies/16S rRNA) and upstream river (3.30 × 10^−4^ gene copies/16S rRNA).

**Table 2:** Concentrations with standard deviations of antibiotic resistance genes in the upstream rivers, WWTP effluent and downstream points at NW-E and NW-C (units in gene copies/16S rRNA ± standard deviation).

Location	Sites	EBC	ACC	CIT	DHA	MOX	FOX	*IntI1*	*Sul1*
NW-E	Up	1.90 × 10^−2^ ± 1.31 × 10^−2^	1.00 × 10^−1^ ± 1.96 × 10^−1^	–	4.15 × 10^−2^ ± 7.68 × 10^−3^	1.36 × 10^−1^ ± 7.68 × 10^−2^	3.20 × 10^0^ ± 6.54 × 10^−2^	6.61 × 10^−4^ ± 1.06 × 10^−3^	1.16 × 10^−2^ ± 1.14 × 10^−2^
	Effluent	1.96 × 10^0^ ± 1.54 × 10^0^	2.90 × 10^−1^ ± 3.42 × 10^−1^	3.71 × 10^−1^ ± 3.09 × 10^−1^	2.90 × 10^−1^ ± 1.50 × 10^−1^	1.82 × 10^1^ ± 4.35 × 10^−1^	1.45 × 10^0^ ± 1.24 × 10^0^	4.93 × 10^−4^ ± 1.67 × 10^−4^	7.51 × 10^−2^ ± 3.33 × 10^−2^
	Down	2.44 × 10^−1^ ± 9.70 × 10^−3^	6.11 × 10^−2^ ± 2.93 × 10^−2^	2.23 × 10^−3^ ± 1.82 × 10^−3^	9.19 × 10^−3^ ± 1.42 × 10^−3^	–	9.70 × 10^−2^ ± 4.27 × 10^−2^	2.40 × 10^−4^ ± 1.61 × 10^−4^	5.67 × 10^−3^ ± 3.04 × 10^−2^
NW-C	Up	1.65 × 10^−1^ ± 1.40 × 10^−1^	1.97 × 10^−1^ ± 2.52 × 10^−1^	–	6.98 × 10^−2^ ± 1.40 × 10^−1^	7.29 × 10^−1^ ± 1.61 × 10^0^	4.06 × 10^0^ ± 5.03 × 10^−2^	3.30 × 10^−4^ ± 2.13 × 10^−4^	3.88 × 10^−2^ ± 3.19 × 10^−2^
	Effluent	2.87 × 10^0^ ± 5.18 × 10^0^	8.34 × 10^−3^ ± 7.76 × 10^−3^	1.50 × 10^−1^ ± 2.76 × 10^−1^	5.98 × 10^−1^ ± 5.66 × 10^−1^	1.58 × 10^0^ ± 9.37 × 10^0^	7.57 × 10^−1^ ± 1.42 × 10^−2^	4.09 × 10^−4^ ± 1.91 × 10^−4^	3.30 × 10^−1^ ± 2.76 × 10^−2^
	Down	1.66 × 10^0^ ± 9.54 × 10^−1^	8.61 × 10^−2^ ± 1.56 × 10^−2^	4.28 × 10^−1^ ± 4.16 × 10^−1^	1.31 × 10^−1^ ± 5.43 × 10^−2^	3.83 × 10^0^ ± 1.12 × 10^−1^	1.38 × 10^0^ ± 1.90 × 10^−2^	5.38 × 10^−4^ ± 8.38 × 10^−^	3.16 × 10^−2^ ± 2.47 × 10^−2^

Up, upstream river; Effluent, WWTP effluent; down, downstream river for NW-E, downstream maturation pond for NW-C; LOD, limit of detection; ND, not done; “-”not detected by end-point PCR.

At the NW-C WWTP, the downstream maturation pond (4.28 × 10^−1^ gene copies/16S rRNA) had higher ACC abundance than WWTP effluent (1.50 × 10^−1^ gene copies/16S rRNA). In NW-E, the ACC abundance was higher in the WWTP effluent (3.71 × 10^−1^ gene copies/16S rRNA) compared to the downstream river (NW-E = 2.23 × 10^−3^ gene copies/16S rRNA). Since the CIT gene was not detected in the NW-E and NW-C upstream rivers during the end-point PCR screening process, it was not subjected to real-time PCR.

The findings of the present study show that the EBC gene concentrations were higher in the WWTP effluent (NW-E = 1.96 × 10^0^ gene copies/16S rRNA and NW-C = 2.87 × 10^0^ gene copies/16S rRNA) compared to the upstream river (NW-E = 1.90 × 10^−2^ gene copies/16S rRNA and NW-C = 1.65 × 10^−1^ gene copies/16S rRNA) and downstream point (NW-E = 2.44 × 10^−1^ gene copies/16S rRNA and NW-C = 1.66 × 10^0^ gene copies/16S rRNA).

In NW-C, the MOX abundance was higher in the downstream maturation pond (3.83 × 10^0^ gene copies/16S rRNA) than in the WWTP effluent (1.58 × 10^0^ gene copies/16S rRNA) and upstream river (7.29 × 10^−1^ gene copies/16S rRNA). In NW-E, the MOX abundance was higher in the WWTP effluent (1.82 × 10^1^ gene copies/16S rRNA) than in the upstream river (1.36 × 10^−1^ gene copies/16S rRNA).

At both NW-E and NW-C WWTPs, DHA and *sul1* abundance were higher in the WWTP effluents than in the upstream rivers and downstream sampling points (Table 2). The trends observed at the NW-E were as follows; upstream river (DHA = 4.15 × 10^−2^ gene copies/16S rRNA and *sul1 =* 1.16 × 10^−2^ gene copies/16S rRNA), WWTP effluent (DHA = 2.90 × 10^−1^ gene copies/16S rRNA and *sul1 =* 7.51 × 10^−2^ gene copies/16S rRNA) and downstream river (DHA = 9.19 × 10^−3^ gene copies/16S rRNA and *sul1* = 5.67 × 10^−3^ gene copies/16S rRNA). At NW-C, the trends were as follows; upstream river (DHA = 6.98 × 10^−2^ gene copies/16S rRNA and *sul1 =* 3.88 × 10^−2^ gene copies/16S rRNA), WWTP effluent (DHA = 5.98 × 10^−1^ gene copies/16S rRNA and *sul1 =* 3.30 × 10^−1^ gene copies/16S rRNA) and downstream river (DHA = 1.31 × 10^−1^ gene copies/16S rRNA and *sul1* = 3.16 × 10^−2^ gene copies/16S rRNA).

### 3.5 Quantification of antibiotic residues and fluconazole

In NW-E, ampicillin concentrations were higher in WWTP effluent (24.9 μg/L) than in the upstream (12.19 μg/L) and downstream rivers (22.68 μg/L), as shown in Table 3. However, in NW-C, ampicillin concentrations were higher in the WWTP effluent to the downstream maturation pond. The upstream river had a lower concentration (Table 4). In NW-C, ciprofloxacin concentrations in WWTP effluent (1.51 μg/L) and downstream maturation pond (1.50 μg/L) were comparable, with the concentration in the upstream river being lower (0.83 μg/L; Table 4). Furthermore, in NW-E, higher concentrations of the contaminant were measured in the wastewater effluent than in upstream and downstream river samples (Table 3). At the NW-C WWTP, sulfamethoxazole concentrations were lower in the WWTP effluent (0.98 μg/L) and downstream maturation pond (0.34 μg/L), compared to the upstream river site (1.22 μg/L; Table 4). In NW-E, the sulfamethoxazole concentrations in upstream (2.49 μg/L) and downstream rivers (2.50 μg/L) were comparable, but higher compared to wastewater effluent (5.06 μg/L; Table 3).

**Table 3:** Concentrations with standard deviations of target fluconazole and antibiotic residues in NW-E WWTP effluent, upstream and downstream rivers.

Date	Sampling sites	Ampicillin (μg/L)	Ciprofloxacin (μg/L)	Sulfamethoxazole (μg/L)	Trimethoprim (μg/L)	Fluconazole (μg/L)
Oct-20	Up	14.46 ± 6.32	3.06 ± 0.13	3.36 ± 0.12	< LOQ	< LOQ
	Effluent	11.97 ± 1	4.03 ± 0.34	3.51 ± 0.13	< LOQ	< LOQ
	Down	ND	ND	ND	ND	ND
Nov-20	Up	16.86 ± 2.23	0.59 ± 0.17	4.04 ± 0.28	< LOQ	< LOQ
	Effluent	21.35 ± 1.85	0.53 ± 0.07	3.87 ± 0.09	< LOQ	< LOQ
	Down	ND	ND	ND	ND	ND
Dec-20	Up	14.26 ± 4.85	0.42 ± 0.08	3.33 ± 1.22	< LOQ	< LOQ
	Effluent	24.94 ± 0.11	1.96 ± 0.50	3.82 ± 0.04	< LOQ	< LOQ
	Down	ND	ND	ND	ND	ND
Feb-21	Up	17.81 ± 1.48	2.38 ± 0.13	3.85 ± 0.20	< LOQ	< LOQ
	Effluent	22.72 ± 1.24	4.44 ± 0.66	3.66 ± 0.06	< LOQ	< LOQ
	Down	ND	ND	ND	ND	ND
Jul-21	Up	6.67 ± 0.38	1.38 ± 0.21	< LOQ	0.21 ± 0.01	< LOQ
	Effluent	37.77 ± 2.15	2.17 ± 0.37	< LOQ	0.7 ± 0.14	1.16 ± 0.06
	Down	ND	ND	ND	ND	ND
Aug-21	Up	3.93 ± 0.34	< LOQ	< LOQ	< LOQ	< LOQ
	Effluent	30.25 ± 1.52	< LOQ	1.97 ± 0.17	0.41 ± 0.08	< LOQ
	Down	ND	ND	ND	ND	ND
Feb-22	Up	11.37 ± 2.28	1.00 ± 0.13	2.86 ± 0.23	0.20 ± 0.02	6.60 ± 1.61
	Effluent	25.30 ± 6.69	< LOQ	18.57 ± 0.63	< LOQ	24.77 ± 0.81
	Down	22.68 ± 1.12	< LOQ	2.5 ± 0.45	< LOQ	17.3 ± 0.92

Up, upstream river; Effluent, WWTP effluent; down, downstream river; LOQ, limit of quantification; ND, not done; ± − standard deviation.

**Table 4:** Concentrations with standard deviations of target fluconazole and antibiotic residues in NW-C upstream rivers, WWTP effluent, and downstream maturation pond at NW-C.

Date	Sampling sites	Ampicillin (μg/L)	Ciprofloxacin (μg/L)	Sulfamethoxazole (μg/L)	Trimethoprim (μg/L)	Fluconazole (μg/L)
Nov-20	Up	15.10 ± 4.75	< LOQ	< LOQ	< LOQ	< LOQ
	Effluent	15.80 ± 1.86	0.77 ± 0.14	3.74 ± 0.03	0.24 ± 0.02	< LOQ
	Down	16.34 ± 0.45	1.04 ± 0.03	3.39 ± 0.40	0.3 ± 0.02	< LOQ
Dec-20	Up	10.56 ± 2.66	2.25 ± 0.56	2.36 ± 0.78	< LOQ	< LOQ
	Effluent	16.83 ± 2.95	2.78 ± 0.11	3.83 ± 0.12	0.32 ± 0.01	< LOQ
	Down	12.06 ± 1.10	2.38 ± 0.30	3.77 ± 0.01	0.73 ± 0.2	< LOQ
Feb-21	Up	11.29 ± 0.86	1.87 ± 0.17	3.76 ± 0.22	< LOQ	< LOQ
	Effluent	12.64 ± 0.02	3.71 ± 1.39	3.75 ± 0.11	0.15 ± 0.02	< LOQ
	Down	20.94 ± 2.56	2.01 ± 0.33	3.77 ± 0.05	< LOQ	< LOQ
Jul-21	Up	7.36 ± 0.61	< LOQ	< LOQ	0.21 ± 0.02	< LOQ
	Effluent	9.19 ± 0.68	0.53 ± 0.06	1.16 ± 0.13	4.70 ± 0.34	< LOQ
	Down	8.08 ± 0.98	1.66 ± 0.01	< LOQ	0.43 ± 0.03	< LOQ
Aug-21	Up	8.54 ± 1.33	< LOQ	< LOQ	< LOQ	< LOQ
	Effluent	12.37 ± 2.9	0.46 ± 0.04	6 ± 0.78	0.48 ± 0.06	1.3 ± 0.05
	Down	13.99 ± 1.11	0.43 ± 0.05	< LOQ	0.23 ± 0.01	< LOQ
Feb-22	Up	–	–	–	–	–
	Effluent	3.35 ± 0.76	0.81 ± 0.01	< LOQ	< LOQ	31.62 ± 2.82
	Down	–	–	–	–	–

Up, upstream river; Effluent, WWTP effluent; down, downstream maturation pond; LOQ, limit of quantification.

In NW-E, trimethoprim and fluconazole concentrations were often below the LOQ for river and WWTP effluent samples (Table 3). The LOQs for trimethoprim and fluconazole were 0.13 μg/L and 1.01 μg/L, respectively. In February 2022, higher fluconazole concentrations were measured in the upstream river (6.60 μg/L), WWTP effluent (24.77 μg/L) and downstream river (17.30 μg/L) compared to other months. Higher trimethoprim concentrations in the upstream river (0.21 μg/L) and WWTP effluent (0.70 μg/L) were measured in samples collected in July 2021.

In NW-C, similar observations were made in the upstream river and WWTP effluent, where fluconazole concentrations were often below the LOQ (Table 4). Higher fluconazole concentrations were measured in WWTP effluent samples (31.62 μg/L) in February 2022. Furthermore, higher trimethoprim concentrations were recorded in the WWTP effluent, except in November and December 2020, where higher concentrations were measured in the downstream maturation pond (Table 4).

### 3.6 Risk quotient of WWTP effluents and rivers

**Figure 5:**
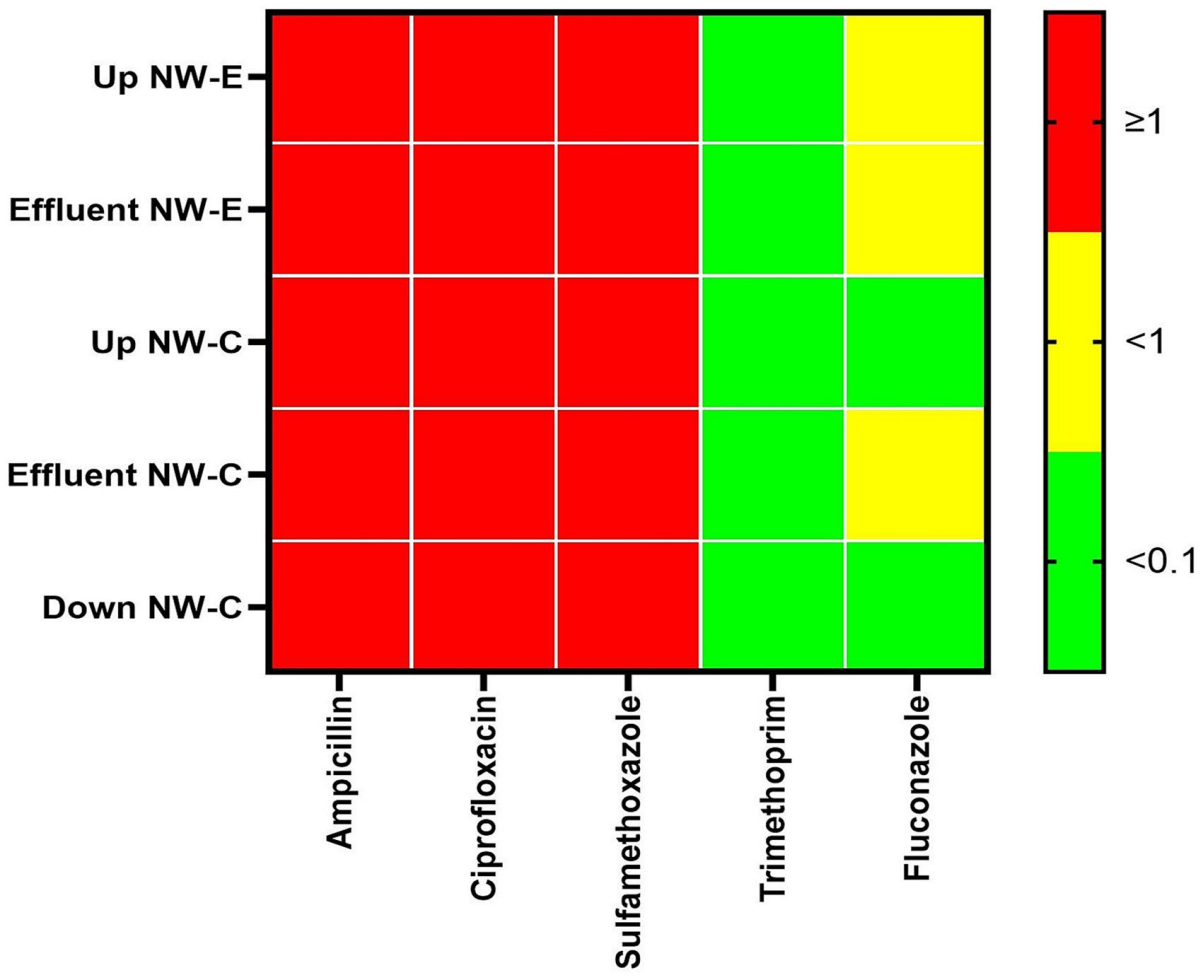
Heatmap of risk quotient (RQ) in the upstream rivers, WWTP effluent and downstream points at NW-E and NW-C. Up, upstream river; Effluent, WWTP effluent; down, downstream maturation pond.

The higher risk was associated with high levels of ampicillin, ciprofloxacin and sulfamethoxazole (RQ ≥ 1). The lowest risk was associated with the levels of trimethoprim, as determined in all samples, where RQ < 0.1 was observed (Figure 5). Similar observations were made for fluconazole in the NW-C upstream river and the downstream maturation pond. Furthermore, NW-E upstream river and WWTP effluent, as well as NW-C WWTP effluent, recorded fluconazole RQs < 1, indicating a medium risk.

### 3.7 Correlation of bacterial communities with ARGs, antibiotics and physicochemical parameters

**Figure 6:**
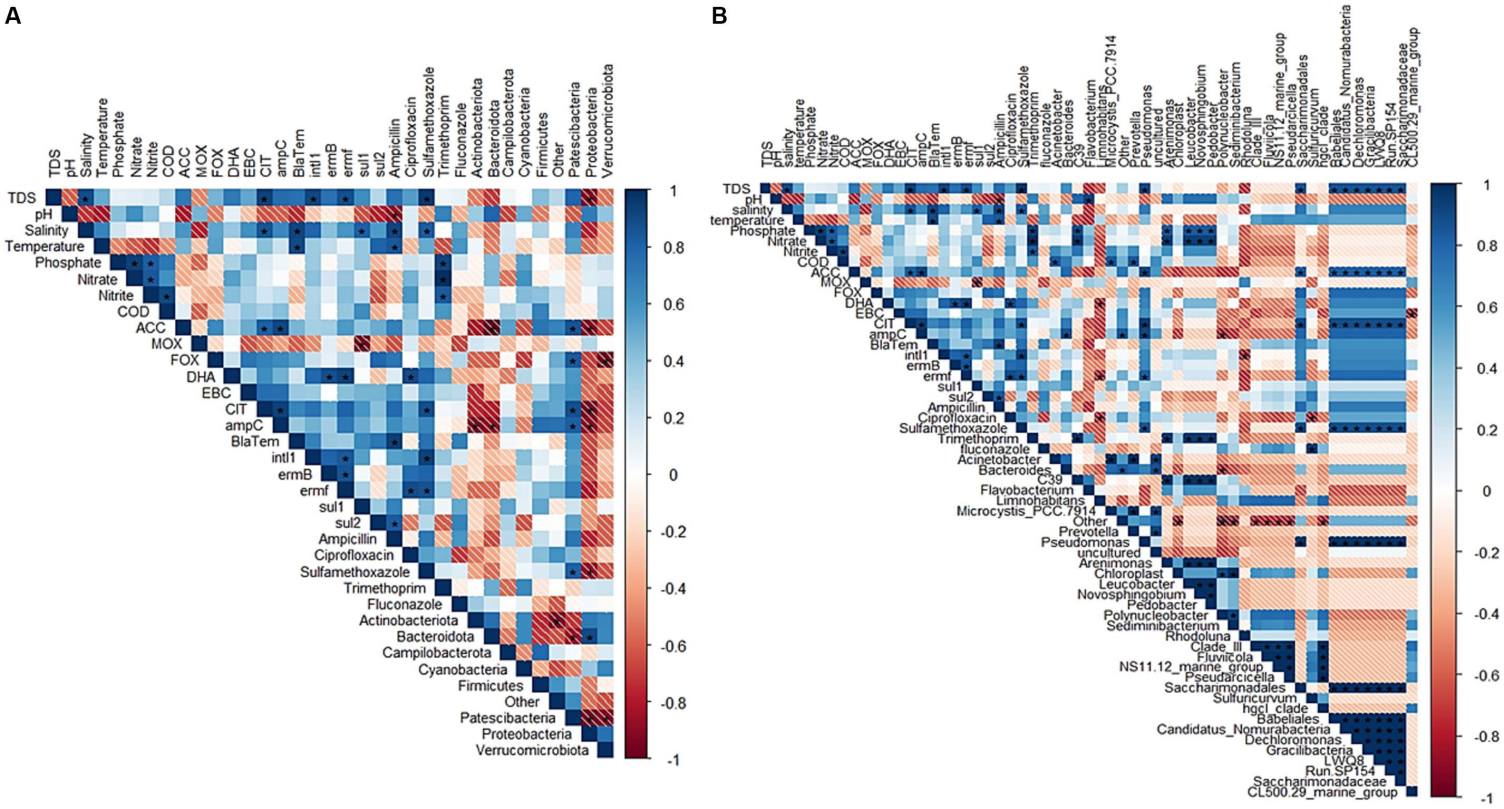
Correlation plot with significant levels between bacterial community composition [**(A)** phyla- and **(B)** genera], physicochemical parameters and antibiotic residues in the upstream rivers, wastewater effluent and downstream points at NW-E and NW-C. Up, upstream river; Effluent, WWTP effluent; down, downstream maturation pond.

Figure 6 depicts a correlation plot with significant levels between bacterial communities with ARGs, antibiotic residues and physicochemical parameters. In this section, the focus was only on the significant correlation (*p*-value < 0.05) of bacterial communities with antibiotic residues, physicochemical parameters, and ARGs, and the trend observed for phyla in Figure 6A was as follows: (i) Actinobacteriota significantly correlated with the *ampC* gene. (ii) Bacteroidota significantly correlated with *ampC* and MOX genes. (iii) Patescibacteria significantly correlated with ACC, DHA, CIT, *ampC genes*, and sulfamethoxazole. (iv) Proteobacteria significantly correlated with TDS, ACC, CIT, and sulfamethoxazole. (v) Verrucomicrobiota significantly correlated FOX gene.

Figure 6B shows correlation patterns at the genus level. Similar correlation patterns were observed for genera *Saccharimonadales, Babeliales, Candidatus Nomurabacteria*, *Dechloromonas, Gracilibacteria*, LWQ8, Run-SP154 and *Saccharimonadaceae*. Significant correlations for these genera were reported for TDS, ACC, CIT and sulfamethoxazole. Moreover, *Arenimonas*, C39, *Leucobacter, Novosphingobium* and *Pedobacter* showed similar correlation patterns and were significantly correlated with nitrate, nitrite and trimethoprim.

## 4 Discussion

The present study recorded physicochemical parameters in WWTP effluents and rivers. This data filled the gap in the literature during a seven-year period when the DWS did not publish the Green Drop report. The physicochemical parameters reported in NW-E and NW-C WWTP effluents are of utmost importance to establish the chemical compliance of these WWTPs. The DWS (2022) reports that 92% of monitored WWTPs in North West, South Africa, were deemed to be of critical risk.

Overall, the TDS and salinity levels of NW-E were higher than those obtained in NW-C. This may be due to mining activities in the catchment of NW-E. Furthermore, higher TDS and salinity levels in the downstream maturation pond compared to wastewater effluent in NW-C may be associated with raw sewage leakage and the disposal of litter directly into the maturation pond. However, these were not in accordance with other studies, which reported higher TDS (Pillay and Olaniran, 2016; Ngigi et al., 2020) and salinity levels (Nthunya et al., 2018) in the upstream rivers compared to the WWTP effluent.

Consistent with NW-C, Pillay and Olaniran (2016) and Ofori et al. (2022) reported higher phosphate levels in the WWTP effluent, followed by the downstream point and upstream river. However, this was inconsistent with NW-E, where higher phosphate levels were recorded in the downstream river, which may be associated with agricultural activities occurring in and around this site. The COD levels (ranging from 20 to 45 mg/L) in NW-E were comparable to those measured in the Czech Republic (Ofori et al., 2022) but lower than those measured in South African studies (Pillay and Olaniran, 2016; Adefisoye and Okoh, 2017).

In NW-E, insignificantly (*p*-value > 0.05) higher nitrate levels in WWTP effluent (3.27 mg/L) than in the upstream (0.88 mg/L) and the downstream (1.07 mg/L) rivers were following the results reported by Ofori et al. (2022) in the Czech Republic. However, in NW-C, the nitrate levels in the upstream river (1.14 mg/L) were significantly lower than those in the WWTP effluent (13.37 mg/L) and the downstream maturation pond (5.35 mg/L). This is common since surface water is cleaner than WWTP effluent. According to the WHO (2016), the present study findings indicate that WWTP effluent and surface water were not contaminated with nitrate. The low nitrite level in the NW-E downstream river may be misleading due to the insufficient number of sampling replicates. In NW-C, comparable nitrite levels were measured in the WWTP effluent (19.11 mg/L) and downstream maturation pond (19.17 mg/L).

Consistent with other studies, maximum and minimum temperatures were measured in the summer and winter months (Adefisoye and Okoh, 2017; Nthunya et al., 2018). The pH measured in NW-E and NW-C is similar to that reported in other South African WWTPs and surface water (Nthunya et al., 2018; Makuwa et al., 2020). Furthermore, in NW-C, significant differences (*p*-value < 0.05) in pH were observed between the upstream river and the downstream maturation pond, which may be due to raw sewage spillage and littering by residents of NW-C into the downstream maturation pond. Overall, according to the DWAF (1996a,b) for agricultural use (livestock watering and irrigation), water from NW-E and NW-C is suitable for agriculture. However, abnormalities were reported for pH levels above 8.4, which could damage plant foliage. High pH values were anticipated in the NW-E surface water, since this catchment lies on dolomite bedrock rich in calcium and magnesium carbonates (Koekemoer et al., 2021). Moreover, the measured physicochemical parameters may favor the growth of microorganisms in NW-E and NW-C water (WHO, 2022).

Consistent with other STUDIES, the present study reported a high abundance of the phylum Proteobacteria as the dominant group (Tang et al., 2016; Wang et al., 2018; Osunmakinde et al., 2019; Pascual-Benito et al., 2020). Consistent with Pascual-Benito et al. (2020), the present study reported a high relative abundance of the phylum Bacteroidota. Studies reported that species belonging to this group are associated with faecal pollution and some may be pathogenic (Osunmakinde et al., 2019; Pascual-Benito et al., 2020). Species belonging to the Actinobacteriota phylum can be used to monitor water quality, since they are sensitive towards environmental conditions that cause cyanobacterial blooms (Pascual-Benito et al., 2020).

Similar results to those of Tang et al. (2016) were reported in the present study, where the relative abundance of Verrucomicrobiota in some rivers was higher compared to WWTPs. Verrucomicrobiota is associated with the degradation of stable polysaccharides (Osunmakinde et al., 2019). The phylum Firmicutes is a good indicator of faecal pollution (Wang et al., 2018), as demonstrated in the Daspoort WWTP, Pretoria, South Africa (Osunmakinde et al., 2019).

In NW-E, comparable species diversity, dominance and richness in rivers and the WWTP effluent may be attributed to recreational, anthropogenic, agricultural and mining activities occurring in and around these sources (Tang et al., 2016; Coertze and Bezuidenhout, 2020). In NW-C, the downstream maturation pond, which passes through a residential area, was prone to sewage leakage, litter, and livestock farming. Hence, it was more diverse, richer, even and dominant, but with no significant difference compared to the WWTP effluent and upstream water.

As indicated, NW-E and NW-C water harbor potential human, animal and plant pathogens that may be spread through the food chain. This could lead to high morbidity and mortality in humans and animals. Furthermore, the use of such contaminated water could harm economically important crops (Dobhal et al., 2020). As shown in the section below, these potential and opportunistic pathogens may harbor ARGs.

The presence of ARGs in the aquatic environment poses a public health threat due to their potential spread to clinically relevant opportunistic pathogens. A wide range of ARGs belonging to *β*-lactamase, macrolide, sulfonamide and mobile genetic elements were detected in NW-E and NW-C WWTPs and surrounding rivers. The results provide in-depth insights into the impact of aquatic environments as reservoirs of antibiotic resistance in South Africa, a region still underexplored in global antimicrobial resistance surveillance efforts.

The present study’s findings show that the detection of *sul1* and *sul2* genes, which confer resistance to sulfonamide, increased from the WWTP effluents to the downstream points in NW-E and NW-C. This increase may be attributed to agricultural activities occurring in and around downstream points in NW-E and NW-C. Huang et al. (2021) report sulfonamide’s excretion in cattle and swine manure. Once in the environment, sulphonamides can persist in manure with a half-life of eight to 30 days (Boxall et al., 2004; Massé et al., 2014). Furthermore, in NW-C, anthropogenic activities and sewage leakage contribute to the increased detection of these genes in the downstream maturation pond. The presence of *sul1* and *sul2* genes in WWTPs and rivers was reported in KwaZulu-Natal, South Africa (Suzuki et al., 2015). The occurrence of these genes in the upstream rivers, without the influence of WWTPs, supports that they are associated with anthropogenic activities (Freeman et al., 2018).

The presence of *bla_TEM_*, *ampC*, and pAmpCs in NW-E and NW-C reflects a diverse *β*-lactam resistance gene pool and may be attributed to the use of *β*-lactam antibiotics in livestock farming. *β*-lactams may be spread in water environments through excretion, where they can persist for a short time with a half-life of 5 days (Boxall et al., 2004; Massé et al., 2014). A study reported that 133.000 μg/kg of *β*-lactams were detected in the manure of swine (weighing 150 pounds on average) in a confined feeding operation (Huang et al., 2021).

Potential pathogenic bacteria encoding pAmpC *β*-lactamase confer resistance to third-generation cephalosporins (Estaleva et al., 2021; Matloko et al., 2021). Thus, the detection of clinically relevant pAmpCs, associated with HGT, indicates a potential spread of resistomes between environmental and clinical settings (Coertze and Bezuidenhout, 2019). Estaleva et al. (2021) detected pAmpCs resistance genes in clinical isolates of *Escherichia coli* (25/230) isolated from urine and blood cultures at Maputo Central Hospital, Mozambique. Furthermore, *Escherichia coli* isolates (24/65) encoding pAmpC resistance genes were reported from clinical environments, cattle faeces, beef products, human faeces and borehole water in North-West, South Africa (Matloko et al., 2021). As supported by Coertze and Bezuidenhout (2019), it is not unusual to detect these genes in environmental samples in the North West Province, South Africa. The application of water-harboring *β*-lactam resistance genes for domestic and agricultural activities may facilitate the spread of these genes to drinking water and crops (Tsholo et al., 2024; Richter et al., 2021). This highlights that water is a vector of antibiotic resistance and as such, this environment should be explored more to combat antibiotic resistance.

The detection of the *ermB* gene in 60% of NW-C upstream river and NW-E downstream river samples was comparable to that reported in Pearl River and Pearl River Estuary, South China (Chen et al., 2015). However, the presence of these genes in environmental water is poorly documented in South Africa, underscoring the importance of regional surveillance efforts. The detection of *ermB* and *ermF* genes may be enhanced by the poor metabolism of associated antibiotics, resulting in 50%–90% excretion of macrolides and 60% excretion of lincosamides (Quaik et al., 2020). The presence of the *ermB* and *ermF* genes in NW-E and NW-C may contribute to the selection of macrolide resistance. The NDoH (2021) report indicates that this antibiotic is essential for both human and animal use. The application of macrolides is well documented by the Africa CDC (2021). This antibiotic (azithromycin) can be used in situations where a patient has a medical contraindication, confirmed or suspected dysentery and can also be used in African regions where cholera is endemic or outbreaks are occurring. The co-occurance of *ermB* and *ermF* with other ARGs, as shown in NW-E and NW-E, suggests that there are multi-antimicrobial resistance clusters and selective pressure from continuous use of antibiotics in human and animal health (NDoH, 2021).

In NW-E and NW-C, the detection of the *intI1* gene was higher, ranging from 60% to 100%. This is not uncommon, as Thakali et al. (2020) reported in a WWTP in Louisiana, USA, and Chen et al. (2015) in the Pearl River and Pearl River Estuary, South China. The presence of the *intI1* gene in NW-E and NW-C water could enable its host bacteria to induce multidrug resistance (Chen et al., 2015). The *intI1* gene is used as a gene marker for anthropogenic activities and is commonly detected in environments contaminated with antibiotics (Freeman et al., 2018). Furthermore, the high detection levels of the *intI1* gene and ARGs in the WWTP effluent and surrounding water may be linked to the abundance of available nutrients and biofilm formation (Thakali et al., 2020).

Furthermore, the present study determined the abundance of *sul1*, *intI1*, and pAmpCs in NW-E and NW-C. The searched databases revealed that Coertze and Bezuidenhout (2020) were the only authors who determined the abundance of pAmpCs in South African surface water. However, no study addressed the influence of WWTP and maturation ponds on receiving water bodies. Thus, the present study is of utmost importance.

In NW-E, the abundance of CIT and EBC genes increased from the WWTP effluent to the downstream river. However, this was not the case for FOX, DHA, MOX and ACC genes. Higher abundance of FOX was higher in the upstream river (3.20 × 10^0^ gene copies/16S rRNA) than in WWTP effluent (1.45 × 10^0^ gene copies/16S rRNA) and downstream river (9.70 × 10^−2^ gene copies/16S rRNA). In NW-C, a higher abundance of DHA and EBC was reported in WWTP effluent, followed by the downstream maturation pond and upstream river. However, the abundance of FOX and ACC genes was higher in the upstream river and lower in the WWTP effluent. This may be attributed to urbanization. Increasing MOX and CIT abundance from the WWTP effluent to the downstream maturation pond may be associated with littering, agricultural activities and sewage leakage.

Inconsistent with Freeman et al. (2018), higher *sul1* abundance in NW-E was measured in the upstream river than in the WWTP effluent and downstream rivers. The reasons for the increase are outlined in the section above. Furthermore, in NW-C, the increase of *sul1* concentration from the WWTP effluent to the downstream maturation may be attributed to the abovementioned reasons. In NW-E and NW-C water samples, *sul1* abundance was present at 10^−3^–10^−1^ gene copies/16S rRNA and was comparable to 10^−2^–10^−1^ gene copies/16S rRNA reported in urban rivers, rural rivers and WWTPs in Durban, South Africa (Suzuki et al., 2015). However, a higher *intI1* abundance was measured in the WWTP effluent in NW-E and NW-C. The *intI1* abundance was lower than that reported in Wascana Creek, Canada (Freeman et al., 2018).

Higher abundance of ARGs in the NW-E and NW-C WWTP effluent comes as no surprise since WWTPs are not designed to remove ARGs (Chamkal et al., 2021; Späth et al., 2021). The discharge of this water into receiving water bodies may affect the microbial community, allowing opportunistic bacteria and pathogens to harbor and spread ARGs (Proia et al., 2016). The application of contaminated water may further spread ARGs to humans and animals. However, no risk assessment tools were developed to determine the risks associated with ARGs (Proia et al., 2016). In addition, ARGs in water are not routinely monitored anywhere in the world, thus making water a vector for antibiotic resistance.

A general trend was observed in NW-E and NW-C, where antibiotic and fluconazole residues were higher in the WWTP effluent compared to the rivers. However, in NW-C, the ampicillin concentrations were higher in the downstream maturation pond (14.28 μg/L) than in the WWTP effluent (11.70 μg/L) and upstream river (10.57 μg/L). This increase in the downstream maturation pond may be attributed to sewage spillage and anthropogenic activities occurring in and around the pond. Furthermore, South Africa has a higher usage of broad-spectrum penicillin, almost 1.3 to 3.3 times higher than countries with advanced public health (NDoH, 2018); hence, the widespread use of ampicillin in NW-E and NW-C water. These findings highlight the One Health relevance of aquatic environments for antimicrobial surveillance and underscore the urgent need for antibiotic stewardship to preserve the effectiveness of antimicrobials.

According to the NDoH (2018), there was a 398% increase in the procurement of trimethoprim from 2014 to 2015 in South Africa. This resulted in the procurement of 175,049 kg of trimethoprim (including sulfonamides) in the country. However, the obtained trimethoprim concentrations in the NW-E and NW-C were low. The reason for this could have been established if there had been antibiotic data for each province. However, trimethoprim concentrations in WWTP effluents of the present study were comparable to those reported by Späth et al. (2021) in Newlands Mash WWTP effluent collected in eThekwini, South Africa. Overall, sulfamethoxazole residues were present in NW-E and NW-C, with a few exceptions. This was anticipated, since animal bodies poorly metabolize this antibiotic, leading to excretion into the environment (Quaik et al., 2020). The presence of sulfamethoxazole residues in the environment may contribute to the development of resistance, as indicated by the presence of *sul1* and *sul2* genes. Sulfamethoxazole and trimethoprim are used in combination to treat *Pneumocystis pneumonia* in patients infected with HIV (Africa Centres for Disease Control and Prevention (CDC), 2021).

The reason for the spike in fluconazole in NW-E and NW-C in February, 2022 could not be established due to a lack of available antifungal information in South Africa. These spikes in concentrations were higher than those reported in a study conducted in Gauteng, South Africa (Monapathi et al., 2021). However, in NW-E and NW-C, the fluconazole concentrations were < LOQ for most sampling months. This was in accordance with results obtained by Monapathi et al. (2021) in North West, South Africa. High sulfamethoxazole concentrations in NW-E and NW-C may be attributed to its use in South Africa (NDoH, 2018). The application of ciprofloxacin in South African agriculture is common. According to the NDoH (2018), a total of 6,907 kg of fluoroquinolones, a class to which ciprofloxacin belongs, were used for animals. In human health, ciprofloxacin inhibits the synthesis of topoisomerase IV and DNA gyrase (Richards et al., 2019).

Additionally, the present study utilized antibiotic and fluconazole concentrations to calculate the RQ. Sulfamethoxazole, ampicillin, and ciprofloxacin were deemed to be of high risk. The high risk of ciprofloxacin concurs with results reported in Morocco and Kenya (Kairigo et al., 2020; Chamkal et al., 2021). High-risk sulfamethoxazole was reported in Juliaca downstream river, Peru (Nieto-Juárez et al., 2021). The high-risk ampicillin follows those reported in the hospital WWTP in Morocco (Chamkal et al., 2021). The risk of fluconazole ranged from low to medium risk in NW-E and NW-C, which was within the results reported in Gauteng, South Africa (Assress et al., 2020). Consistent with Peru’s Puno and Juliaca WWTP effluents, the present study reported a low risk of trimethoprim (Nieto-Juárez et al., 2021).

The spread of antibiotic and fluconazole residues into receiving water bodies through the WWTP effluent was anticipated since WWTPs were not designed for their removal. The presence of antibiotic and fluconazole residues may favor the proliferation of antimicrobial resistance (Chen et al., 2015; Monapathi et al., 2021). Once in the environment, antibiotic and fluconazole residues pose ecological risks. Despite this, antibiotic and fluconazole residues are not routinely monitored in water environments as part of the water quality assessment. As a result, environmental studies such as these are typically not considered in policymaking efforts aimed at mitigating antibiotic resistance. The effects of antibiotic resistance may be severe in South Africa, where there is a high number of immunocompromised individuals and yet the health care is deteriorating (Statistics South Africa, 2022).

Although the physicochemical parameters were within the recommended standards of DWAF SAWQG (DWAF, 1996a; DWAF, 1996b) for agricultural use, they may create conducive environments and provide nutrients needed for the survival of opportunistic and pathogenic bacteria. In NW-E and NW-C, the interaction between opportunistic and pathogenic bacteria and antibiotic residues in sub inhibitory concentrations may select for antibiotic resistance. This results in the emergence of ARGs that are harbored and shared between opportunistic and pathogenic bacteria for survival.

The correlation analysis was applied to investigate the potential relationship between bacterial communities with ARGs, antimicrobials and physicochemical parameters. *Saccharimonadales, Babeliales, Candidatus Nomurabacteria, Dechloromonas*, *Gracilibacteria*, LWQ8, Run-SP154 and *Saccharimonadaceae* exhibited a significant correlation with ACC and CIT genes, suggesting that these genera may serve as possible reservoirs of *β*-lactam resistance (cephalosporin). They also show possible enrichment under sulfamethoxazole pressure in the environment. Meanwhile, *Arenimonas*, C39, *Leucobacter*, *Novosphingobium,* and *Pedobacter* thrive in NW-E and NW-C nitrate and nitrite environments. The significant correlation between bacterial communities and antibiotic residues is worrisome, since it reflects a potential adaptation to antimicrobials, which may exert selective pressures that promote the emergence of antibiotic resistance (Boxall et al., 2004; Massé et al., 2014).

The findings of NW-E and NW-C suggest that WWTPs are unable to completely remove antimicrobial residues and ARGs. The presence of genes associated with mobile genetic elements promotes the spread of ARGs to opportunistic bacteria in the environment (Thakali et al., 2020). Given the high levels of antibiotic resistance, there is an urgent need for the development and introduction of alternative antimicrobial approaches. Mitigation strategies for antimicrobial resistance require routine monitoring of antimicrobial residues and ARGs in water quality assessments. This could aid in establishing water quality guidelines and standardized risk methods for antimicrobial residues and ARGs.

While this present study adds to the body of knowledge on ARGs and antimicrobial residues on WWTPs and surrounding water, it has limitations. Wastewater influent samples were not analysed and as a result, it is impossible to determine whether the two WWTPs were able to reduce the levels of contaminants. Furthermore, a limited number of ARGs, one antifungal and no antifungal resistance genes were investigated.

## 5 Conclusion

In the South African context, the present study contributed by (i) determining ARG abundance in WWTPs and their receiving water bodies and (ii) reporting the chemical water quality when the DWS was inconsistent with the publication of Green Drop reports. The present study also contributes to the growing body of knowledge on utilizing culture-independent techniques and high-throughput 16S rRNA sequencing to investigate bacterial communities.

The present study’s findings showed that bacterial communities, physicochemical parameters, ARGs, antibiotic and fluconazole residues are present in NW-E and NW-C. Correlation established that the prevalence of bacterial communities is associated with physicochemical parameters, ARGs, and antimicrobial residues. However, despite the evidence that antibiotics fluconazole and ARGs may pose health risks to animals, humans and the environment, experts have paid no heed to their surveillance and management in water environments. As a result, strategies to combat antibiotic resistance have been associated with clinical settings, but the present study proves that environmental settings are as important. Thus, the present study’s findings are of utmost importance in policymaking to improve water quality, antibiotic use and tackling antibiotic resistance.

## Data Availability

The datasets presented in this study can be found in online repositories. The names of the repository/repositories and accession number(s) can be found in the article/Supplementary material.

## Footnotes

^1^ https://data.qiime2.org/2023.9/common/silva-138-99-nb-classifier.qza
