# Supplementary material for: Bacterial community composition, quantification of antibiotic resistance genes and antibiotic residues in wastewater treatment plant and receiving rivers
Source: Front Microbiol. 2025 Oct 27;16:1635253. doi: 10.3389/fmicb.2025.1635253 (PMC12599842; doi:10.3389/fmicb.2025.1635253)
Supplement: Supplementary file 1 [file Data_Sheet_1.docx]

Table S1: Oligonucleotide primers for the end-point PCR amplification of *sul1* and s*ul2* *bla_TEM_, ampC, intI1, ermB* and *ermF* genes.

| Target gene | Primer's name | Sequence (5'…..3') | Size  (bp) | PCR conditions | References |
| --- | --- | --- | --- | --- | --- |
| *ampC* | AmpC-F | TTCTATCAAMACTGGCARCC | 550 | 5 min at 94ºC, 35 cycles = 5 min at 94ºC 30 s at 49ºC, 1 min at 72ºC and 7 min at 72 ºC | Coertze and Bezuidenhout, 2017 |
|  | AmpC-R | CCYTTT TATGTACCCAYGA |  |  |  |
| *bla_TEM_* | TEM-F | ATTCTTGAAGACGAAAGGGC | 1 150 | 5 min at 94ºC, 30 cycles = 5 min at 95ºC, 1 min at 60ºC, 1 min at 72ºC and 5 min at 72ºC | Costa *et al*., 2007 |
|  | TEM-R | ACGCTCAGTGGAACGAAAAC |  |  |  |
| *IntI1* | HS463A | CTGGATTTCGATCACGGCACG | 473 | 5 min at 94ºC, 30 cycles = 30 s at 94ºC, 30s at 64ºC, 1 min at 72ºC and 5 min at 72 ºC | Labbate *et al.,* 2008 |
|  | HS464 | ACATGCGTGTAAATCATCGTCG |  |  |  |
| *ermB* | ermB-F | GAAAAGGTACTCAACCAAATA | 638 | 5 min at 95ºC, 35 cycles = 30 s at 95ºC, 1 min at 48ºC, 1 min at 72ºC and 5 min at 72ºC | Tran *et al.,* 2013 |
|  | ermB-R | AGTAACGGTACTTAAATTGTTTAC |  |  |  |
| *ermF* | ermF1 | CGGGTCAGCACTTTACTATTG | 466 | 5 min at 95ºC, 35 cycles = 30s at 95ºC, 30s at 50ºC, 2 min at 72ºC and 10 min at 72ºC | Chung *et al.,* 1999 |
|  | ermF2 | GGACCTACCTCATAGACAAG |  |  |  |
| *Sul1* | SulI-F | TTCGGCATTCTGAATCTCAC | 822 | 3 min at 94ºC, 35 cycles = 1 min at 94ºC, 1 min at 60ºC, 2 min at 72ºC and 5 min at 72 ºC | Chaturvedi *et al*., 2021 |
|  | SulI-R | ATGATCTAACCCTCGGTCTC |  |  |  |
| *Sul2* | SulII-F | CGGCATCGTCAACATAACC | 722 | 3 min at94ºC, 35 cycles =1 min at 94ºC, 1 min at 60ºC, 1 min at 72ºC and 5 min at 72 ºC | Chaturvedi *et al*., 2021 |
|  | SulII-R | GTGTGCGGATGAAGTCAG |  |  |  |

F- Forward primer and R- Reverse primer

Table S2: Oligonucleotide primers for the end-point PCR amplification of six pAmpCs, namely MOX, EBC, ACC, FOX, DHA, and CIT genes.

| **Target gene** | **Primer's name** | **Sequence (5'…..3')** | **Size**  **(bp)** | **PCR conditions** | **References** |
| --- | --- | --- | --- | --- | --- |
| *ACC* | ACCMF | AACAGCCTCAGCAGCCGGTTA | 346 | 3 min at 94ºC, 35 cycles = 30s at 94ºC, 30s at 64ºC, 1 min at 72ºC and 7 min at 72ºC | Pérez-Pérez and Hanson, 2002 |
|  | ACCMR | TTCGCCGCAATCATCCCTAGC |  |  |  |
| *MIR-1T ACT-1* | EBCMF | TCGGTAAAGCCGATGTTGCGG | 302 | 3 min at 94ºC, 35 cycles = 30s at 94ºC, 30s at 64ºC, 1 min at 72ºC and 7 min at 72ºC |  |
|  | EBCMR | CTTCCACTGCGGCTGCCAGTT |  |  |  |
| *FOX-1 to FOX-5b* | FOXMF | AACATGGGGTATCAGGGAGATG | 190 | 3 min at 94ºC, 35 cycles = 30s at 94ºC, 30s at 64ºC, 1 min at 72ºC and 7 min at 72ºC |  |
|  | FOXMR | CAAAGCGCGTAACCGGATTGG |  |  |  |
| *DHA-1, DHA-2* | DHAMF | AACTTTCACAGGTGTGCTGGGT | 405 | 3 min at 94ºC, 35 cycles = 30s at 94ºC, 30s at 64ºC, 1 min at 72ºC and 7 min at 72ºC s |  |
|  | DHAMR | CCGTACGCATACTGGCTTTGC |  |  |  |
| *LAT-1 to LAT-4, CMY-2 to CMY-7, BIL-1* | CITMF | TGGCCAGAACTGACAGGCAAA | 462 | 3 min at 94ºC, 35 cycles = 30s at 94ºC, 30s at 62ºC, 1 min at 72ºC and 7 min at 72ºC |  |
|  | CITMR | TTTCTCCTGAACGTGGCTGGC |  |  |  |
| *MOX-1, MOX-2, CMY-1, CMY-8* | MOXMF | GCTGCTCAAGGAGCACAGGAT | 520 | 3 min at 94ºC, 35 cycles = 30s at 94ºC, 30s at 59ºC, 1 min at 72ºC and 7 min at 72ºC |  |
|  | MOXMR | CACATTGACATAGGTGTGGTGC |  |  |  |

F- Forward primer and R- Reverse primer

Table S3: Oligonucleotide primers for the real-time PCR quantification of the *IntI1*, *sul1* and 16S rRNA genes.

| **Target genes** | **Primer's name** | **Sequence (5'…..3')** | **PCR conditions** | **References** |
| --- | --- | --- | --- | --- |
| 16S rRNA | 906F | AAACTCAAAKGAATTGACGG | Hold stage:2 min at 50ºC. PCR stage: 5 min at 95ºC, 40 cycles = 15 s at 95ºC, 15 s at 55ºC and 15 s at 72ºC | Passera *et al.*, 2021 |
|  | 1062R | CTCACRRCACGAGCTGAC |  |  |
| *sul1* | FW | CGCACCGGAAACATCGCTGCAC | Hold stage:2 min at 50ºC. PCR stage: 5 min at 95ºC, 40 cycles = 15 s at 95ºC, 30 s at 60ºC and 15 s at 72ºC | Xu *et al.,* 2017 |
|  | RV | TGAAGTTCCGCCGCAAGGCTCG |  |  |
| *IntI1* | FW | GGCTTCGTGATGCCTGCTT | Hold stage:2 min at 50ºC. PCR stage: 5 min at 95ºC, 40 cycles = 15 s at 95ºC, 30 s at 55ºC and 15 s at 72ºC | Xu *et al.,* 2017 |
|  | RV | CATTCCTGGCCGTGGTTCT |  |  |

F-forward primer and R-reverse primer

Table S4: Method validation parameters for quantifying ARGs and 16S rRNA.

|  | Y-intercept | Slope | Efficiency | R^2^ | Ct cutoff |
| --- | --- | --- | --- | --- | --- |
| DHA | 41.62 | -3.46 | 95.00 | 0.999 | 37.14 |
| ABC | 44.43 | -3.22 | 104.54 | 0.999 | 26.60 |
| ACC | 40.38 | -3.45 | 95.03 | 0.991 | 39.48 |
| CIT | 39.06 | -3.44 | 95.24 | 0.994 | 36.64 |
| FOX | 41.01 | -3.26 | 102.61 | 0.990 | 39.06 |
| IntI1 | 27.77 | -3.34 | 99.23 | 0.990 | 26.47 |
| MOX | 41.05 | -3.36 | 98.32 | 0.985 | 38.95 |
| Sul1 | 25.67 | -3.42 | 96.06 | 0.991 | 24.99 |
| 16S rRNA | 22.95 | -3.25 | 102.93 | 0.985 | 25.62 |

R^2^ = coefficient of determination

Table S5: Method validation parameters for quantifying antibiotic and fluconazole residues.

| **Target residues** | **Mass spectrometry (m/z)** | **Retention time (min)** | **LOD** (**µg/L)** | **LOQ** (**µg/L)** | ***R*^2^** | **Intra-day precision** (**%RSD)** | | | **Inter-day precision** (**%RSD)** | **Accuracy** (**%)** |
| --- | --- | --- | --- | --- | --- | --- | --- | --- | --- | --- |
|  |  |  |  |  |  | **Day 1** | **Day 2** | **Day 3** |  |  |
| **Ampicillin** | 350.1500 | 3.300 | 0.24 | 0.79 | 0.999 | 5 | 4 | 5 | 5 | 90 |
| **Trimethoprim** | 291.1473 | 3.181 | 0.04 | 0.13 | 0.994 | 3 | 1 | 3 | 11 | 101 |
| **Ciprofloxacin** | 332.1446 | 3.464 | 0.11 | 0.36 | 0.998 | 4 | 2 | 3 | 13 | 102 |
| **Sulfamethoxazole** | 254.0588 | 9.200 | 0.34 | 1.14 | 0.997 | 6 | 9 | 2 | 20 | 93 |
| **Fluconazole** | 307.111 | 5.007 | 0.30 | 1.01 | 0.999 | 5 | 2 | 1 | 19 | 98 |

R^2^ = coefficient of determination; LOD = limit of detection; LOQ= Limit of quantification; RSD = relative standard deviation

Table S6: 16S rRNA metabarcoding sample names and their corresponding accession numbers

| number | Sample ID | Accession no: | Sample | Location | Repository URL |
| --- | --- | --- | --- | --- | --- |
| 1 | KT1_S42_L001 | SRR33684884 | Downstream river | NWE | <https://www.ncbi.nlm.nih.gov/bioproject?LinkName=sra_bioproject&from_uid=38798239> |
| 2 | KT2_S43_L001 | SRR33684883 | Upstream river | NWE | <https://www.ncbi.nlm.nih.gov/bioproject?LinkName=sra_bioproject&from_uid=38798239> |
| 3 | KT3_S44_L001 | SRR33684872 | Wastewater effluent | NWE | <https://www.ncbi.nlm.nih.gov/bioproject?LinkName=sra_bioproject&from_uid=38798239> |
| 4 | KT4_S45_L001 | SRR33684870 | Downstream river | NWE | <https://www.ncbi.nlm.nih.gov/bioproject?LinkName=sra_bioproject&from_uid=38798239> |
| 5 | KT5_S46_L001 | SRR33684869 | Upstream river | NWE | <https://www.ncbi.nlm.nih.gov/bioproject?LinkName=sra_bioproject&from_uid=38798239> |
| 6 | KT6_S47_L001 | SRR33684868 | Wastewater effluent | NWE | <https://www.ncbi.nlm.nih.gov/bioproject?LinkName=sra_bioproject&from_uid=38798239> |
| 7 | KT7_S48_L001 | SRR33684867 | Downstream maturation pond | NWC | <https://www.ncbi.nlm.nih.gov/bioproject?LinkName=sra_bioproject&from_uid=38798239> |
| 8 | KT8_S49_L001 | SRR33684866 | Upstream river | NWC | <https://www.ncbi.nlm.nih.gov/bioproject?LinkName=sra_bioproject&from_uid=38798239> |
| 9 | KT9_S50 | SRR33684865 | Wastewater effluent | NWC | <https://www.ncbi.nlm.nih.gov/bioproject?LinkName=sra_bioproject&from_uid=38798239> |
| 10 | KT10_S51_L001 | SRR33684864 | Downstream maturation pond | NWC | <https://www.ncbi.nlm.nih.gov/bioproject?LinkName=sra_bioproject&from_uid=38798239> |
| 11 | KT11_S52_L001 | SRR33684882 | Upstream river | NWC | <https://www.ncbi.nlm.nih.gov/bioproject?LinkName=sra_bioproject&from_uid=38798239> |
| 12 | KT12_S53_L001 | SRR33684881 | Wastewater effluent | NWC | <https://www.ncbi.nlm.nih.gov/bioproject?LinkName=sra_bioproject&from_uid=38798239> |

**References**

1. Chaturvedi, P., Singh, A., Chowdhary, P., Pandey, A. and Gupta, P. (2021). Occurrence of emerging sulfonamide resistance (*sul1* and *sul2*) associated with mobile integrons-integrase (*intI1* and *intI2*) in riverine systems. *Science of the Total Environment*, 751:142217. DOI: [10.1016/j.scitotenv.2020.142217](https://doi.org/10.1016/j.scitotenv.2020.142217)
2. Chung, W.O., Werckenthin, C., Schwarz, S., Roberts, M.C. (1999). Host range of the *ermF* rRNA methylase gene in bacteria of human and animal origin. *Journal of Antimicrobial Chemotherapy*, 43:5-14. DOI: [10.1093/jac/43.1.5](https://doi.org/10.1093/jac/43.1.5)
3. Coertze, R.D. and Bezuidenhout, C.C. (2017). The prevalence and diversity of *AmpC* β-lactamase genes in plasmids from aquatic systems. *Water Science and Technology*, 2:1-9. DOI: [10.2166/wst.2018.188](https://doi.org/10.2166/wst.2018.188)
4. Costa, D., Poeta, P., Sáenz, Y., Coelho, A.C., Matos, M., Vinué, L., et al. (2007). Prevalence of antimicrobial resistance and resistance genes in faecal isolates recovered from healthy pets. *Veterinary Microbiology*, 127:97-105. DOI: [10.1016/j.vetmic.2007.08.004](https://doi.org/10.1016/j.vetmic.2007.08.004)
5. Labbate, M., Chowdhury, P.R. and Stokes, H.W. (2008). A Class 1integron present in a human commensal has a hybrid transposition module compared to tn402: evidence of interaction with mobile DNA from natural environments. *Journal of Bacteriology*,190(15):5318–5327. DOI: [10.1128/JB.00199-08](https://doi.org/10.1128/JB.00199-08)
6. Passera, A., Follador, A., Morandi, S., Miotti, N., Ghidoli, M., Venturini, G., et al. (2021). Bacterial communities in the embryo of maize landraces: Relation with susceptibility to Fusarium Ear Rot. *Microorganisms*, *9*(11), p.2388. <https://doi.org/10.3390/microorganisms9112388>
7. Pérez-Pérez, F.J. and Hanson, N.D. (2002). Detection of plasmid-mediated *AmpC* β-lactamase genes in clinical isolates by using multiplex PCR. *Journal of Clinical Microbiology*, 40(6):2153-2162.  DOI: [10.1128/JCM.40.6.2153-2162.2002](https://doi.org/10.1128/JCM.40.6.2153-2162.2002)
8. Tran, C.M., Tanaka, K. and Watanabe, K. (2013). PCR-based detection of resistance genes in anaerobic bacteria isolated from intra-abdominal infections. Journal of Infection and *Chemotherapy*, 19(2):279-90. DOI: [10.1007/s10156-012-0532-2](https://doi.org/10.1007/s10156-012-0532-2)
9. Xu, Y., Xu, J., Mao, D. and Luo, Y. (2017). Effect of the selective pressure of sub-lethal level of heavy metals on the fate and distribution of ARGs in the catchment scale. *Environmental Pollution*, 220:900-908. DOI: [10.1016/j.envpol.2016.10.074](https://doi.org/10.1016/j.envpol.2016.10.074)
